# Induction of systemic, mucosal and memory antibody responses targeting *Vibrio cholerae* O1 O-specific polysaccharide (OSP) in adults following oral vaccination with an oral killed whole cell cholera vaccine in Bangladesh

**DOI:** 10.1371/journal.pntd.0007634

**Published:** 2019-08-01

**Authors:** Aklima Akter, Pinki Dash, Amena Aktar, Sultana Rownok Jahan, Sadia Afrin, Salima Raiyan Basher, Al Hakim, Asura Khanam Lisa, Fahima Chowdhury, Ashraful I. Khan, Peng Xu, Richelle C. Charles, Meagan Kelly, Pavol Kováč, Jason B. Harris, Taufiqur Rahman Bhuiyan, Stephen B. Calderwood, Edward T. Ryan, Firdausi Qadri

**Affiliations:** 1 icddr,b (International Centre for Diarrhoeal Disease Research, Bangladesh), Dhaka, Bangladesh; 2 NIDDK, LBC, National Institutes of Health, Bethesda, Maryland, United States of America; 3 Division of Infectious Diseases, Massachusetts General Hospital, Boston, Massachusetts, United States of America; 4 Department of Medicine, Harvard Medical School, Boston, Massachusetts, United States of America; 5 Division of Global Health, Massachusetts General Hospital for Children, Boston, Massachusetts, United States of America; 6 Department of Pediatrics, Harvard Medical School, Boston, Massachusetts, United States of America; 7 Department of Microbiology, Harvard Medical School, Boston, Massachusetts, United States of America; 8 Department of Immunology and Infectious Diseases, Harvard School of Public Health, Boston, Massachusetts, United States of America; George Washington University School of Medicine and Health Sciences, UNITED STATES

## Abstract

**Background:**

Oral cholera vaccine (OCV) containing killed *Vibrio cholerae* O1 and O139 organisms (Bivalent-OCV; Biv-OCV) are playing a central role in global cholera control strategies. OCV is currently administered in a 2-dose regimen (day 0 and 14). There is a growing body of evidence that immune responses targeting the O-specific polysaccharide (OSP) of *V*. *cholerae* mediate protection against cholera. There are limited data on anti-OSP responses in recipients of Biv-OCV. We assessed serum antibody responses against O1 OSP, as well as antibody secreting cell (ASC) responses (a surrogate marker for mucosal immunity) and memory B cell responses in blood of adult recipients of Biv-OCV in Dhaka, Bangladesh.

**Methodology/Principal findings:**

We enrolled 30 healthy adults in this study and administered two doses of OCV (Shanchol) at days 0 and 14. Blood samples were collected before vaccination (day 0) and 7 days after each vaccination (day 7 and day 21), as well as on day 44. Serum responses were largely IgA with minimal IgG and IgM responses in this population. There was no appreciable boosting following day 14 vaccination. There were significant anti-OSP IgA ASC responses on day 7 following the first vaccination, but none after the second immunization. Anti-OSP IgA memory B cell responses were detectable 30 days after completion of the vaccination series, with no evident induction of IgG memory responses. In this population, anti-Ogawa OSP responses were more prominent than anti-Inaba responses, perhaps reflecting impact of previous exposure. Serum anti-OSP responses returned to baseline within 30 days of completing the vaccine series.

**Conclusion:**

Our results call into question the utility of the 2-dose regimen separated by 14 days in adults in cholera endemic areas, and also suggest that Biv-OCV-induced immune responses targeting OSP are largely IgA in this highly endemic cholera area. Studies in children in cholera-endemic areas need to be performed. Protective efficacy that extends for more than a month after vaccination presumably is mediated by direct mucosal immune response which is not assessed in this study. Our results suggest a single dose of OCV in adults in a cholera endemic zone may be sufficient to mediate at least short-term protection.

## Author summary

Cholera, which can be a severe watery diarrheal illness caused by the non-invasive bacterium *Vibrio cholerae*, remains a global public health concern for many developing countries. Immune responses targeting the O-specific polysaccharide (OSP) of *V*. *cholerae* are involved in mediating protection against cholera. Evidence suggests that inactivated whole-cell oral cholera vaccine (OCV) that includes killed *V*. *cholerae* O1 and O139 organisms provides protection against cholera caused by *V*. *cholerae* O1, but the immune correlates of this protection are not fully defined. In this study, we specifically assessed induction of immune responses targeting *V*. *cholerae* O1 OSP following vaccination with the OCV in adults in a cholera endemic area, Bangladesh. Our results show that OCV induces serum, mucosal and memory anti-OSP responses, and that these responses are maximal following the first dose of vaccination, without evident boosting with the second dose. This result calls into question the day 0 and day 14 two dose regimen currently used among adults in a cholera-endemic zone.

## Introduction

Cholera, a dehydrating diarrheal illness that is caused by the bacterium *Vibrio cholerae*, remains a leading public health problem for many of the world’s impoverished individuals [1]. More than 200 serogroups of *V*. *cholerae* have been characterized to date, but in most recent years serogroups O1 has been the cause of epidemic cholera [2, 3]. Based on genotypic and phenotypic differences, the O1 serogroup can be divided into two phenotypically distinct classical and El Tor biotypes, and both biotypes into Ogawa and Inaba serotypes [4]. The classical biotype is no longer identified, and the El Tor biotype is now the circulating strain of *V*. *cholerae* O1; the prevalent serotype of El Tor *V*. *cholerae* O1 often fluctuates between and even during cholera outbreaks, switching between Ogawa and Inaba [5]. The difference between these two serotypes is determined by the absence of a 2-*O*-methyl group in the Inaba type in the terminal carbohydrate of the O-specific polysaccharide (OSP) in the lipopolysaccharide (LPS) moiety [6, 7]. Evidence suggests that protection against cholera is serogroup specific and immune responses targeting *V*. *cholerae* OSP, the primary determinant of LPS antigen specificity, may play a central role in mediating such protection [2, 3, 8].

Among the three World Health Organization (WHO) pre-qualified and commercially available OCVs, our primary focus for this study was on Shanchol (Shantha Biotechnics, India), which is an inactivated bi-valent whole cell vaccine containing both biotypes and serotypes of *V*. *cholerae* O1, as well as *V*. *cholerae* serogroup O139 organisms [9]. Previously in a trial conducted in Dhaka, Bangladesh, the currently recommended two-dose regimen (on day 0 and day 14) of the Shanchol vaccine was found to confer 53% protection from symptomatic cholera over 2 years of follow-up [10]. Despite the rapidly growing use of oral cholera vaccination with Shanchol, studies evaluating immune responses following vaccination with the vaccine are limited [11–14] compared to studies characterizing immune responses following natural cholera infection or vaccination with Dukoral (WC- rBS; Crucell, Sweden), which contains both inactivated whole cells of *V*. *cholerae* O1 and also the recombinant cholera toxin B subunit [15]. As a result, there remain significant gaps in our understanding of immune responses after vaccination with Shanchol.

In this study, to determine whether vaccination with Shanchol induced significant systemic and mucosal immune responses, we measured vibriocidal antibody responses, antigen specific-plasma antibody responses, and levels of antibody-secreting cell (ASC) responses against *V*. *cholerae* O1 OSP at different time points following vaccination with Shanchol among healthy adult participants in Bangladesh. Memory B cells (MBC) develop following a variety of natural infections and immunizations, and may be a mechanism by which long-term immunity to cholera is mediated [16]. Therefore, we were also interested in assessing whether oral cholera vaccination induced OSP-specific anti-*V*. *cholerae* MBC responses. We present here evidence of the development of systemic and mucosal immune responses to OSP, as well as a circulating OSP-specific IgA MBC population after vaccination.

## Methods and materials

### Ethics statement

Shanchol is pre-qualified by the WHO and is recommended to be used in populations at risk for outbreaks such as Bangladesh. We obtained approval for this study from the Research Review Committee and Ethical Review Committee of the icddr,b and the Institutional Review Board of the Massachusetts General Hospital. Informed written consent was obtained from all participants prior to enrollment.

### Study design and sample collection from participants

A total of 30 adult healthy volunteers were enrolled in this study. Participants were administered two doses of Shanchol at a 14-day interval (at day 0 and day 14). Blood specimens were collected from the participants on day 0 (before vaccination), day 7 (7 days after first vaccination), day 21 (7 days after second vaccination), and day 44. At each time point, vibriocidal antibodies and IgA, IgG, and IgM antibodies in plasma to *V*. *cholerae* O1 Ogawa and Inaba O-specific polysaccharide (OSP) were assessed. We also assessed circulating antigen-specific IgA and IgG ASCs at day 7 and day 21 in isolated peripheral blood mononuclear cells (PBMCs). Antigen (OSP)-specific IgA and IgG MBC levels were measured from PBMCs of vaccinees at day 0 and day 44.

### Isolation of PBMCs and plasma

Sodium-heparinized blood was diluted two-fold with phosphate buffered saline (PBS, pH 7.2–7.4). PBMCs and plasma were separated by density gradient centrifugation using Ficoll-Isopaque (Pharmacia, Piscataway, NJ). Isolated plasma specimens were frozen at– 20^o^ C until used in immunologic assays. PBMCs were re-suspended at a concentration of 1×10^7^ cells/ml in RPMI-complete medium containing RPMI 1640 (Gibco, Carlsbad, CA) and 10% heat-inactivated fetal bovine serum (FBS; HyClone, Logan, UT). The cells were then immediately used for the enzyme-linked immunospot (ELISPOT) assay to determine ASC responses, and cells were also cultured for MBC responses as described below.

### Plasma vibriocidal antibody assay

Vibriocidal antibody responses in plasma at each time point were assessed as previously described using *V*. *cholerae* O1 Ogawa (X-25049) and *V*. *cholerae* O1 Inaba (T-19479) as the target organisms [17]. The vibriocidal titer was defined as the reciprocal of the highest dilution resulting in > 50% reduction of the optical density compared to that of control wells without plasma. Participants were considered as a responder if they had a ≥ 4-fold increase in vibriocidal titer from the baseline level (day 0).

### OSP-specific IgA, IgG, and IgM antibody responses in plasma

Plasma IgA, IgG and IgM antibody responses to OSP were assessed at each time point using standardized protocols of enzyme-linked immunosorbent assays (ELISA), as described previously [2, 12]. In brief, 96 well polystyrene plates (Nunc F, USA) were coated with *V*. *cholerae* O1 Ogawa OSP: BSA (1 μg/ml) or with *V*. *cholerae* O1 Inaba OSP: BSA (1 μg/ml) dissolved in bicarbonate buffer (pH 9.6–9.8). OSP Inaba and Ogawa were generated from *V*. *cholerae* O1 El Tor PIC018 and PIC158, respectively, as previously described [12, 18]. OSP was purified and conjugated to bovine serum albumin (BSA) as previously described [18]. We added 100 μl of plasma (diluted 1:25 in 0.1% BSA in phosphate-buffered saline–0.05% Tween) per well. Horseradish peroxidase-conjugated anti-human IgG or IgA or IgM (Jackson ImmunoResearch, West Grove, PA; 1:1000 dilution) was added to the wells as secondary antibodies, and plates were developed with ortho-phenylene diamine (Sigma, St. Louis, MO) in 0.1 M sodium citrate buffer (pH 4.5) and 0.012% hydrogen peroxide. ELISA plates were read kinetically at 450 nm for 5 minutes and the maximal rate of change in optical density was measured as milli-absorbance units per minute (mabs/min). To monitor inter-assay variability between plates, ELISA units were normalized by calculating the ratio of the test sample to a standard of pooled convalescent-phase plasma from previously infected cholera patients added as a positive control on each plate. Participants were considered as a responder if they had a ≥ 2-fold increase in normalized plasma antibody responses from the baseline level (day 0) [19, 20].

### Detection of antibody-secreting cell (ASC) responses

ASC responses were measured by ELISPOT assay as described previously [21]. To measure the total number of circulating ASCs, nitrocellulose-bottom plates (Millipore, Bedford, MA) were coated with 100 μl of affinity-purified goat anti-human immunoglobulin (Jackson ImmunoResearch, West Grove, PA) at a concentration of 5 μg/ml in PBS. To detect *V*. *cholerae* antigen-specific responses, nitrocellulose plates were also coated with 100 μl of the following antigens: *V*. *cholerae* O1 Ogawa and Inaba OSP: BSA (10 μg/ml), and, as a negative control, keyhole limpet hemocyanin (KLH, Pierce Biotechnology, Rockford, IL, 2.5 μg/ml). To detect IgG and IgA ASCs, alkaline phosphatase-conjugated mouse anti-human IgG (Southern Biotech, Birmingham, AL) and horseradish peroxidase-conjugated IgA (Southern Biotech, Birmingham, AL), each diluted 1:500 in sterile filtered PBS-1%-FBS-tween-0.05%, were added to the plates. Following overnight incubation at 4°C, plates were developed with 5-bromo-4-chloro-3-indolylphosphate-nitroblue tetrazolium (BCIP/NBT, Sigma-Aldrich), and 3-amino-9-ethylcarbazole (AEC pre-mix solution, Sigma-Aldrich), respectively. Two individuals using a stereomicroscope independently quantified IgG and IgA ASC numbers based on the different colored spots. We expressed the number of antigen-specific IgG and IgA ASC as the percentage of the total circulating ASC of the same antibody isotype at the same time point.

### Memory B cell culture and ELISPOT assay

MBC assays were performed using PBMCs on day 0 and day 44 as previously described [2, 13, 22, 23]. 5 x 10^5^ PBMCs/well were placed in cell culture plates (BD Biosciences, San Jose, CA) containing culture media including RPMI 1640, 10% FBS, 2 mM L-glutamine, 200 units/ml penicillin, 200 mg/ml streptomycin, and 50 mM beta-mercaptoethanol. For stimulating antigen-independent proliferation and differentiation of MBCs into ASCs, a mixture of three B-cell mitogens containing 6 μg/ml CpG oligonucleotide (Operon, Huntsville, AL), a 1/100,000 dilution of crude pokeweed mitogen extract, and a 1/10,000 dilution of fixed *Staphylococcus aureus* Cowan (Sigma, St. Louis, MO) was added to all wells except those being used as negative controls, to which only media was added. Culture plates were incubated at 37°C in 5% CO_2_ for 5–6 days; then the cells were harvested and washed. For the MBC ELISPOT assay, ELISPOT plates were coated as above with anti-human immunoglobulin, OSP or KLH and 20% of the cells from each culture plate well were added for detection of total ASC and 80% were used for detection of antigen-specific ASC. These plates were then incubated for 5 hours at 37°C in 5% CO_2_, and the plates were washed, and horseradish peroxidase-conjugated goat anti-human IgA or IgG (Hybridoma Reagent Laboratory, Baltimore, MD) was added at a dilution of 1:500. Plates were incubated overnight at 4°C. On the following day, plates were developed with 3-amino-9-ethyl carbazole (AEC). ELISPOT counts were expressed as the percentage of antigen-specific MBCs out of the total MBCs of that isotype at the same time point. Appropriate stimulation of PBMC was defined as a 3-fold increase in the number of total MBCs after stimulation compared to un-stimulated cells. The limits of detection for responders for antigen-specific IgA and IgG were defined as 0.004% and 0.001%, respectively, per 5x10^5^ PBMC after 6 days of stimulation [13, 24]. We excluded data from analysis for any of the following reasons: (i) the total MBC sample did not have appropriate stimulation, (ii) the study participant’s specimen had four or more antigen-specific ASC spots in the same sample prior to stimulation, or (iii) participant samples had four or more ASC spots to the negative control antigen KLH, as previously described [22, 25].

### Statistical analysis

We assessed the differences in the magnitude of responses within a group using Wilcoxon signed-rank t tests. All reported P values were two-tailed, with a cutoff of P ≤ 0.05 considered a threshold for statistical significance. Seroconversions were compared using the χ^2^ or Fisher’s Exact test. Data analysis and figure preparation were performed using Graphpad Prism 5.0 (GraphPad Software, Inc., La Jolla, CA).

## Results

### Study participants

Thirty healthy adult individuals were enrolled in this study (Table 1). Blood samples were available at all time points for 28 participants. For two individuals, only day 0 and day 44 blood samples were available.

**Table 1:** Demographic characteristics of study participants.

Characteristics		Total (N = 30)
Age, [median (range)]		32 (18–52) years
**Sex**	No. (%) female	19 (63)
	No. (%) male	11 (37)
**No. (%) of participants with blood type:**	O	11 (37)
	A	4 (13)
	B	13 (43)
	AB	2 (7)

### Plasma vibriocidal antibody responses

**Fig 1:**
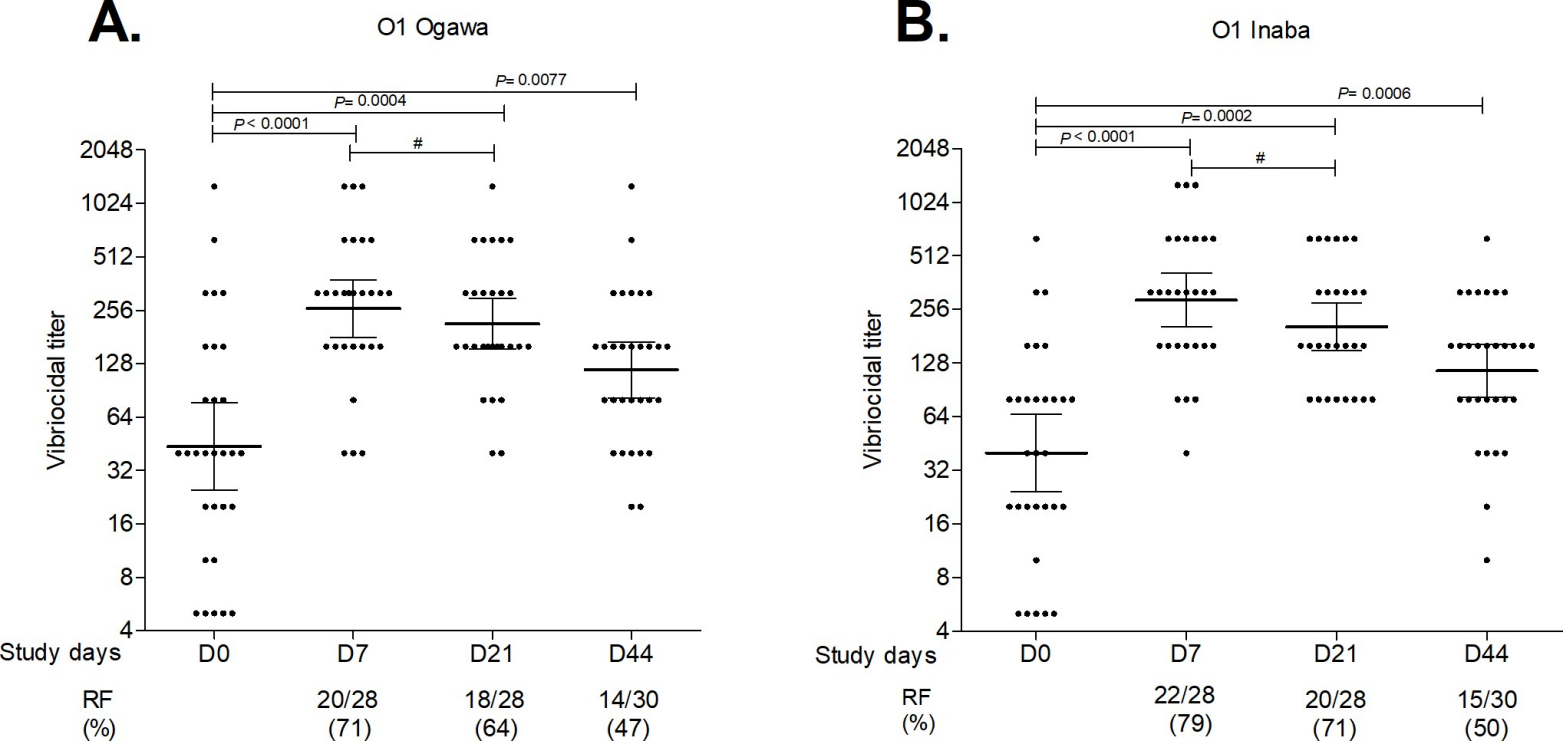
Plasma vibriocidal antibody responses. Geometric mean titer (with 95% CI) of vibriocidal responses to *V*. *cholerae* O1 Ogawa (A) and Inaba (B) in Bangladeshi adults who received two doses of Shanchol vaccine separated by 2 weeks (day 0 and day 14) are represented. Each dot represents an individual vibriocidal antibody titer, horizontal bars represent geometric means (GM) and error bars indicate 95% confidence intervals. The Wilcoxon signed-rank test was used for analyses of the data. P values are indicated where statistically significant differences were obtained from baseline (day 0). Responder frequencies (RF) are also listed. # indicates statistically significant decreases in vibriocidal antibody titers in vaccinees between 7 days after the first and second dose of vaccination (at day 7 and day 21) (P ≤ 0.05).

Fig 1 shows plasma vibriocidal antibody responses in the vaccinees. Significant increases in vibriocidal antibody titers were observed against both Ogawa and Inaba serotypes at all time points compared to baseline (day 0); a robust vibriocidal antibody response was detected by day 7 after a single dose of vaccine (P < 0.0001), which fell thereafter and was not boosted by the second dose of vaccine (Fig 1A and 1B).

Vibriocidal seroconversion was observed at all time points from day 7 onwards, although the highest seroconversion frequency (Ogawa: 71% and Inaba: 79%) was seen at day 7 following the first vaccination. We stratified participants based on their baseline vibriocidal titer and found that those with a baseline (day 0) titer > 80 had a lower seroconversion rate compared to those who had a baseline titer ≤ 80 (Table 2).

**Table 2:** Vibriocidal seroconversion (percentage and number) in adult vaccinees.

	D7/D0		D21/D0		D44/D0	
	Ogawa	Inaba	Ogawa	Inaba	Ogawa	Inaba
All vaccinees [percentage (number)]	71 (20/28)	79 (22/28)	64 (18/28)	71 (20/28)	47 (14/30)	50 (15/30)
Vaccinees with D0 vibriocidal titer ≤ 80 [percentage (number)]	90 (18/20)	91 (21/23)	85 (17/20)	87 (20/23)	64 (14/22)	63 (15/24)
Vaccinees with D0 vibriocidal titer > 80 [percentage (number)]	25 (2/8)	20 (1/5)	13 (1/8)	0 (0/5)	0 (0/8)	0 (0/6)

### *V*. *cholerae* OSP-specific antibody responses in plasma

**Fig 2:**
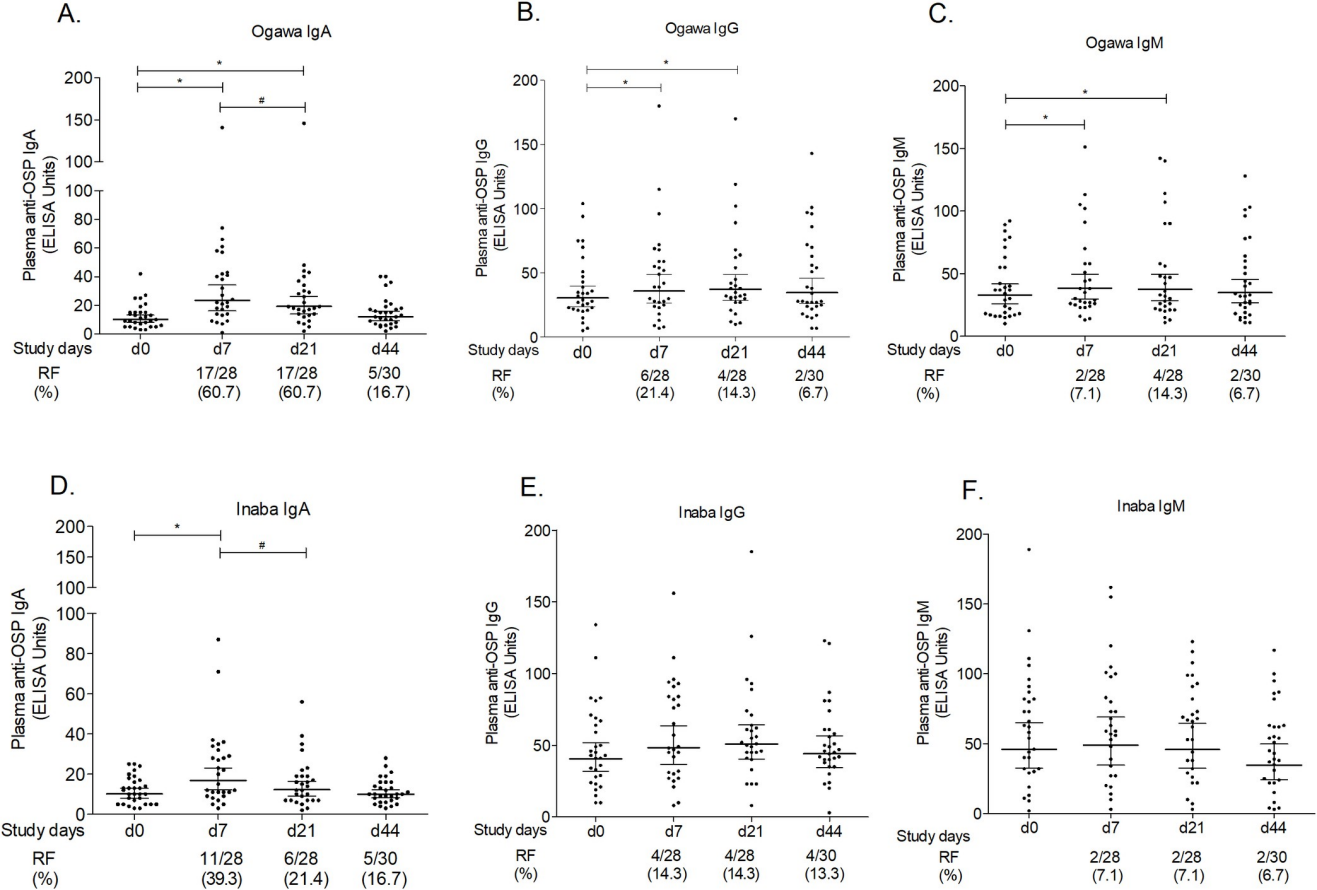
Plasma OSP-specific IgA, IgG and IgM responses. (A) Immunoglobulin A (IgA), (B) IgG, and (C) IgM responses to Ogawa OSP, and (D) IgA, (E) IgG, and (F) IgM responses to Inaba OSP are indicated. Each single dot indicates an individual OSP antibody value; horizontal bars represent geometric means (GM) and error bars indicate 95% confidence intervals. The Wilcoxon signed-rank test was used for analyses of the data. Asterisks indicate a statistically significant difference from baseline level (day 0). Responder frequencies (RF) are shown below the x axes. # indicates a statistically significant decrease in plasma antibody titers in vaccinees 7 days after the first and second dose of vaccination (at day 7 and day 21), respectively (P ≤ 0.05).

We assessed antibody responses to Ogawa and Inaba OSP in plasma. Vaccinees had IgA, IgG and IgM plasma responses to Ogawa OSP that were significantly higher at day 7 and day 21 than the baseline values (P ≤ 0.05) (Fig 2A–2C), but the magnitude of those responses waned by day 44 to baseline levels. Sixty one percent of vaccinees seroconverted at day 7 and day 21 with OSP-specific IgA, but seroconversion in the IgG and IgM isotypes was less (Fig 2A–2C). IgA responses specific for Inaba OSP were also significantly higher at day 7 and day 21 in vaccinees, with a 39% responder frequency at day 7. There was no statistically significant mean increase from the day 0 IgG or IgM responses to Inaba OSP in vaccinees at any subsequent time points (Fig 2D–2F). We did not see any boosting of any of these plasma antibody responses following the second dose of vaccine. We did correlation analysis between plasma antibodies and vibriocidal antibody titers in vaccinees after the first dose of vaccination (at day 7) but no correlation was observed. We also analyzed our data based on baseline vibriocidal titers as above and found that those with a baseline (day 0) titer > 80 had no significant response compared to those who had a baseline titer ≤ 80 (S1 Fig and S2 Fig).

### Circulating IgA antibody-secreting cell (ASC) responses after vaccination

**Fig 3:**
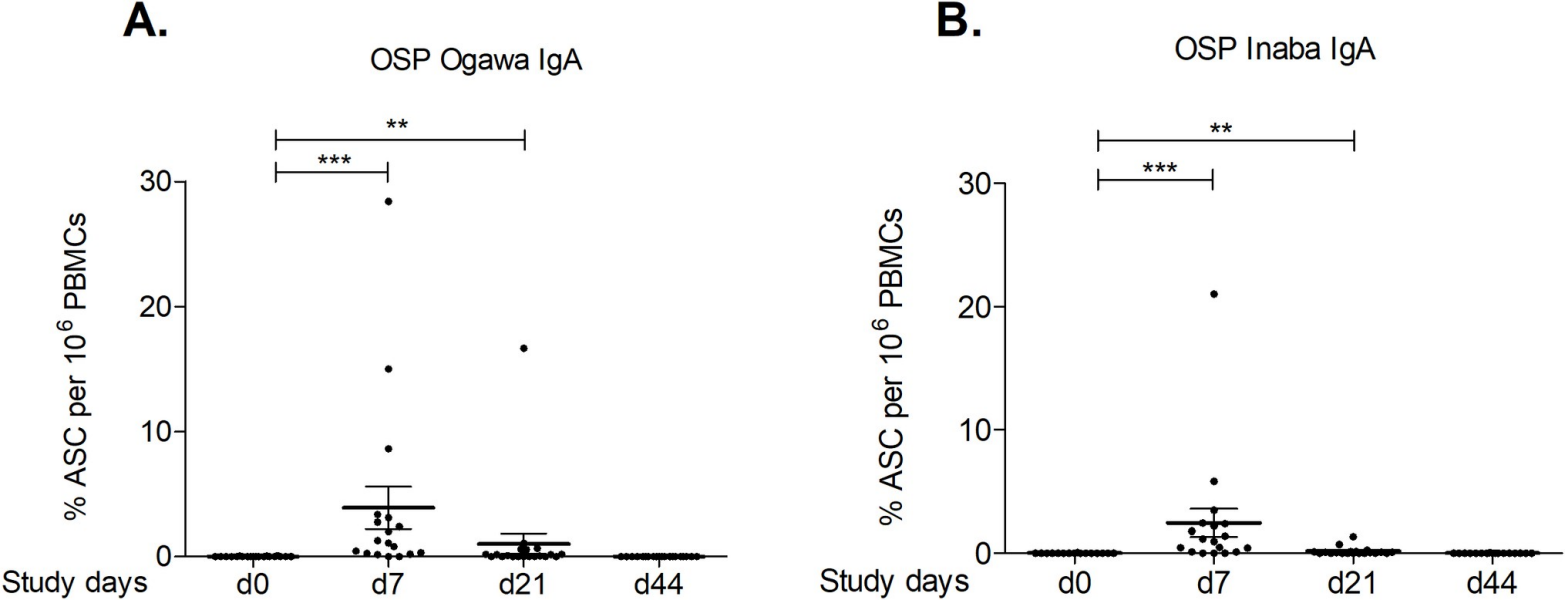
Ogawa and Inaba O-specific polysaccharide (OSP) IgA antibody secreting cell (ASC) responses in vaccine recipients. Mean ± standard errors of the mean (SEM) of the fraction of circulating IgA ASC responses specific for Ogawa (A) and Inaba (B) OSP, out of total circulating IgA ASC responses on the same day. The Wilcoxon signed-rank test was used for analyses of the data. Asterisks denote statistically significant differences from baseline (day 0) (*** = P = 0.0005, ** = P <0.01).

We detected OSP-specific IgA ASC responses in PBMCs from a subset of participants (Fig 3A and 3B). IgA ASC responses specific to Ogawa and Inaba OSP peaked on day 7 following the first dose of vaccine (P = 0.0005) when compared to baseline (day 0). The responses decreased on day 21 but were still significantly elevated compared to baseline; however, we did not see any evidence of boosting of the ASC response with the second dose of vaccine. The levels of IgG ASC responses to OSP were very low and thus were not shown in the figure.

### Antigen-specific IgA and IgG memory B cell (MBC) responses after vaccination

**Fig 4:**
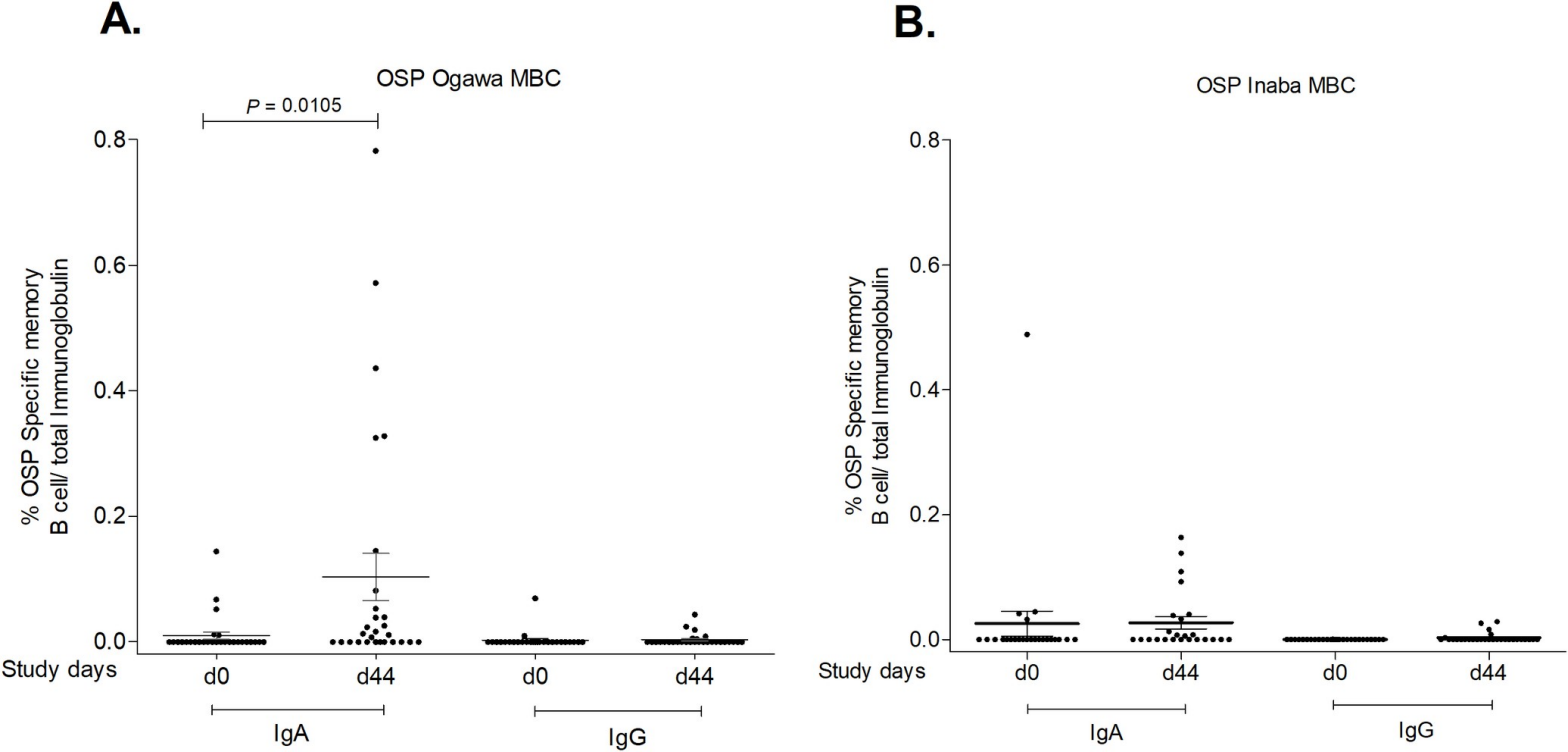
OSP- specific IgA and IgG memory B cell responses. Figure contains mean antigen-specific IgA and IgG memory B cell responses to Ogawa (A) and Inaba (B) OSP, as a percentage of total memory B cells on the same days, with error bars representing standard errors of the means (mean ± SEM). The Wilcoxon signed-rank test was used for analyses of the data. P value is indicated where a statistically significant difference was found from baseline (day 0).

We assessed OSP-specific MBC responses at day 0 and day 44 in vaccinees (Fig 4A and 4B). We detected statistically significant increases in anti-OSP Ogawa-specific IgA MBC responses in day 44 samples compared to day 0 samples (P = 0.0105) (Fig 4A). Detectable IgA MBC responses to Ogawa OSP were found in 17% of vaccinees at day 0 and that increased to 57% at day 44. There was no significant difference in the percentage of Ogawa OSP-specific IgG MBC responses between these two time points. We did not find any statistically significant difference in Inaba OSP-specific IgG or IgA MBCs between day 0 and day 44 (Fig 4B).

## Discussion

This study for the first time characterizes OSP-specific serum, mucosal and memory B cell responses in adults vaccinated with two doses of an oral cholera vaccine, Shanchol, in Bangladesh where it has been shown that OSP-specific serum and mucosal immune responses, as well as IgA memory B cell responses are maximal following the first dose of vaccination, without evident boosting with the second dose.

Cholera has continued to be a significant problem globally, with large scale epidemics in Haiti in 2010 as well as in sub-Saharan Africa [26, 27]. At that time, only a licensed monovalent OCV WC-rBS, called Dukoral has been used, which conferred 85% protective efficacy over the first 6 months following vaccination, but this efficacy decreased to 50% at 3 years in a large field trial in Bangladesh [28]. But after these epidemics, a variety of pilot vaccination campaigns demonstrated the feasibility and effectiveness of the more affordable and easily administered killed whole-cell OCV (Shanchol) in cholera control in Haiti, South Sudan, and Guinea, and this OCV is now considered part of the prevention and control programs in a cholera outbreak [29–31], as in several studies this OCV has shown an overall 66% protective efficacy in a 3-year follow-up to a large field study in Kolkata, India, and a 43% protective efficacy in children under 5 years old [32]. More recently, OCV has been used in large campaigns in a humanitarian crisis in Bangladesh [33]. All killed OCVs are licensed as a two dose regimen with at least a 14-day interval between doses [34], although immunogenicity studies of Shanchol in several countries such as India [35], Haiti [36, 37], Sudan [34], and Philippines [38] have shown little immunologic boosting after the second dose compared to after the first dose of vaccine. Although in some other studies, it has been shown that a single dose of OCV-Shanchol in Bangladesh is 59% protective in older children (>15 years of age) and adults (for at least 2 years) who were presumed to have had pre-existing natural immunity to cholera when vaccinated [39, 40], but there is no evidence that single dose vaccination is good for long term protection and also in children less than 5 years of age.

The serum vibriocidal antibody has previously been the best characterized immunologic correlate of protection against cholera [41, 42]. Vibriocidal antibody is a complement-dependent bactericidal activity resulting largely from IgM antibodies directed against the O-specific component (OSP) of LPS [12, 43–45]. In cholera endemic settings, increasing vibriocidal titers are associated with decreasing risk of cholera, but there is no threshold vibriocidal level at which this protection is complete [36]. We confirmed that OCV in this study resulted in vibriocidal seroconversion in excess of 90% to both serotypes in those individuals whose baseline vibriocidal titer was ≤ 80 on enrollment, and therefore had presumably not recently been infected with *V*. *cholerae* in this endemic area. However, a second dose of vaccine did not provide any immunological boosting, perhaps suggesting that despite having low baseline vibriocidals, many adults in this endemic area had already been primed by previous exposure and had an anamnestic response after first vaccination, so that additional boosting was not seen after the second vaccination.

Because *V*. *cholerae* is a non-invasive pathogen and does not enter the body beyond colonization of intestinal mucosal tissue, protective immunity against this pathogen is more likely mediated by antigen-specific immune responses at the mucosal surface rather than by vibriocidal activity in serum [46]. We have previously shown that individuals infected with *V*. *cholerae* develop prominent serum immune responses to OSP and that these anti-OSP responses, particularly IgM anti-OSP responses, correlate with the serum vibriocidal activity [12]. We have recently shown that the level of pre-existing OSP-specific antibodies in plasma and OSP-specific MBC responses in blood in individuals exposed to cholera in a household with an infected cholera index patient correlate with protection against subsequent cholera in those household contacts [19]. We have also recently shown that OSP-specific serum responses following vaccination with a live oral cholera vaccine correlate with protection against experimental challenge with virulent *V*. *cholerae* O1 in U.S. volunteers [20], though this response might differ from the response of Shanchol. Considering all of these, we were interested in measuring OSP-specific antibody and ASC responses following vaccination in a cholera endemic area following OCV administration. In the present study, we found IgA, IgG, and IgM plasma antibody responses to Ogawa OSP after the first dose of OCV, but again, there was no immunological boosting after the second dose of vaccine. This failure to give immunologic boosting with an OCV at 14 days after the first dose is similar to previous studies that suggested a 14 day interval may be too short to boost immune responses in vaccine recipients in cholera endemic areas [36, 37, 47].

Because cholera is a mucosal infection, gut homing IgA antibody-secreting cell (ASC) responses in blood shortly after infection may also play a role in mediating protective immunity when they return to intestinal tissue [48]. Previous studies have found circulating ASC specific to OSP, LPS and the B subunit of cholera toxin (CTB) at day 7 following natural infection or after vaccination with Dukoral, which then returned to baseline levels [3, 12, 17, 36]. In our current study of Shanchol vaccinees, we also detected IgA OSP-specific circulating ASC 7 days following vaccination, which returned to baseline by day 44; of note, there was no evidence of boosting of mucosal responses after the second dose of vaccine.

Serum and ASC responses following cholera, however, are short-lived [13, 24], suggesting that these short-term responses are not responsible for the longer term protection seen following cholera or cholera vaccination. Instead, the presence of MBCs or long-lived plasma cells to specific cholera antigens may be responsible for mediating long-term protective immunity against cholera [16, 24]. We and others have found prominent MBC responses to OSP, LPS and CTB after natural infection as well as after vaccination with Dukoral, although the duration and magnitude of the MBC responses were lower in vaccinees compared to those following wild-type infection [2, 3, 13, 24]. In our current study, we found significant increases of OSP-specific IgA MBC responses 30 days after completion of the OCV series. Whether these MBC responses to OSP in vaccinees are associated with protection against subsequent disease, as seen in household contacts exposed to cholera, remains to be determined. It will also be informative to assess how long circulating MBCs recognizing the T-cell independent antigen OSP persist in the circulation and/or in mucosal tissues following vaccination. Interestingly, responses seen to Ogawa OSP were much more prominent than to Inaba OSP in our analysis. This may be due to more recent exposure to Inaba serotype organisms in this cholera endemic region, perhaps blunting immune responses. This possibility is supported by the slightly higher mean day 0 values of IgG, IgM and IgA targeting Inaba compared to Ogawa OSP.

Our study has a number of limitations. First, our volunteers were all vaccinated in Bangladesh, a country endemic area for cholera, suggesting many of the vaccinees may have had previous exposure to *V*. *cholerae*. Whether individuals vaccinated with OCV in a non-endemic area would have similar results is not known. Second, all of our participants were adults and how immune responses measured here would differ in children is unknown. Third, we measured ASCs and MBCs in blood and did not directly assess mucosal immune responses, either mucosal memory B cells or long-lived plasma cells, in these volunteers receiving vaccine. Despite these limitations, this is the first study characterizing OSP-specific immune responses in adults receiving two doses of the OCV-Shanchol in Bangladesh and suggests that vaccination produces IgA OSP-specific circulating and mucosal antibody responses as well as MBC responses. Our results call into question the utility of the current two dose vaccination regimen separated by 14 days and support the observation that a single dose of OCV in adults in highly cholera endemic areas may be sufficient to mediate at least short term protection against cholera. For maintaining the long term protection single dose OCV could be administered every year or two. But further studies with more data need to be done to assess the association between immune responses and protection, particularly in children, because children are the most vulnerable population to cholera where the disease is endemic.

## Supporting information

S1 Fig Plasma antibody responses in adult vaccinees with baseline vibriocidal titer < 80 to Ogawa and Inaba O-specific polysaccharide (OSP). Ogawa OSP responses to (A) Immunoglobulin A (IgA), (B) IgG, and (C) IgM and Inaba OSP responses to (D) IgA, (E) IgG, and (F) IgM. Each single dot indicates an individual OSP ELISA unit; horizontal bars represent geometric mean (GM) and error bars indicate 95% confidence intervals. The Wilcoxon signed-rank test was used for analyses of the data. Asterisks indicate a statistically significant difference from baseline level (day 0) (P ≤ 0.05).
(TIF)

S2 Fig Plasma antibody responses in adult vaccinees with baseline vibriocidal titer > 80 to Ogawa and Inaba O-specific polysaccharide (OSP). Ogawa OSP responses to (A) IgA, (B) IgG, and (C) IgM and Inaba OSP responses to (D) IgA, (E) IgG, and (F) IgM. Each single dot indicates an individual OSP ELISA unit; horizontal bars represent geometric mean (GM) and error bars indicate 95% confidence intervals. The Wilcoxon signed-rank test was used for analyses of the data.
(TIF)
