# Supplementary figures and images for: Induction of systemic, mucosal and memory antibody responses targeting Vibrio cholerae O1 O-specific polysaccharide (OSP) in adults following oral vaccination with an oral killed whole cell cholera vaccine in Bangladesh
Source: PLoS Negl Trop Dis. 2019 Aug 1;13(8):e0007634. doi: 10.1371/journal.pntd.0007634 (PMC6692040; doi:10.1371/journal.pntd.0007634)

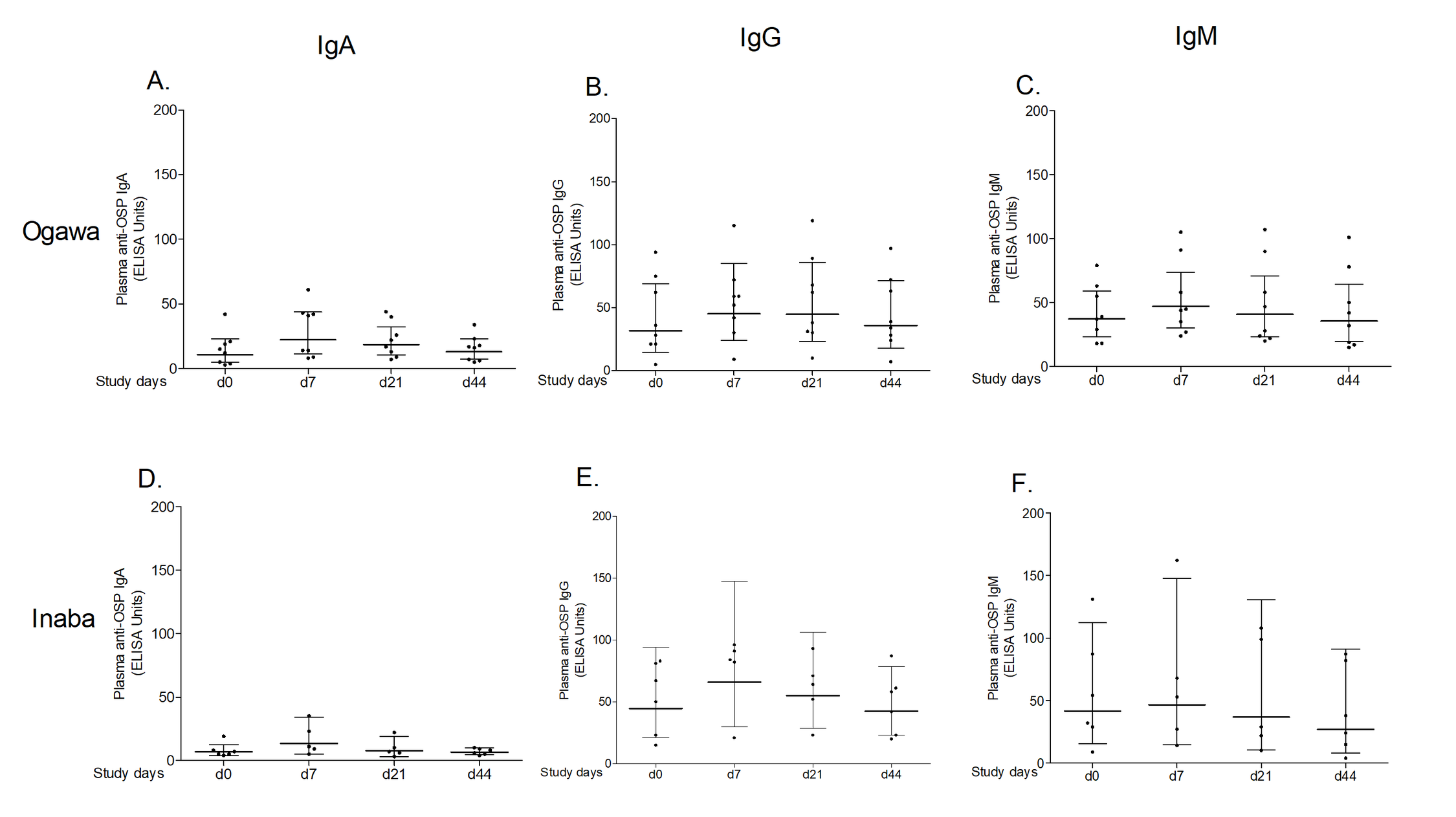

Supplement: S1 Fig — Ogawa OSP responses to (A) Immunoglobulin A (IgA), (B) IgG, and (C) IgM and Inaba OSP responses to (D) IgA, (E) IgG, and (F) IgM. Each single dot indicates an individual OSP ELISA unit; horizontal bars represent geometric mean (GM) and error bars indicate 95% confidence intervals. The Wilcoxon signed-rank test was used for analyses of the data. Asterisks indicate a statistically significant difference from baseline level (day 0) (P ≤ 0.05). (TIF) [file pntd.0007634.s001.tif]

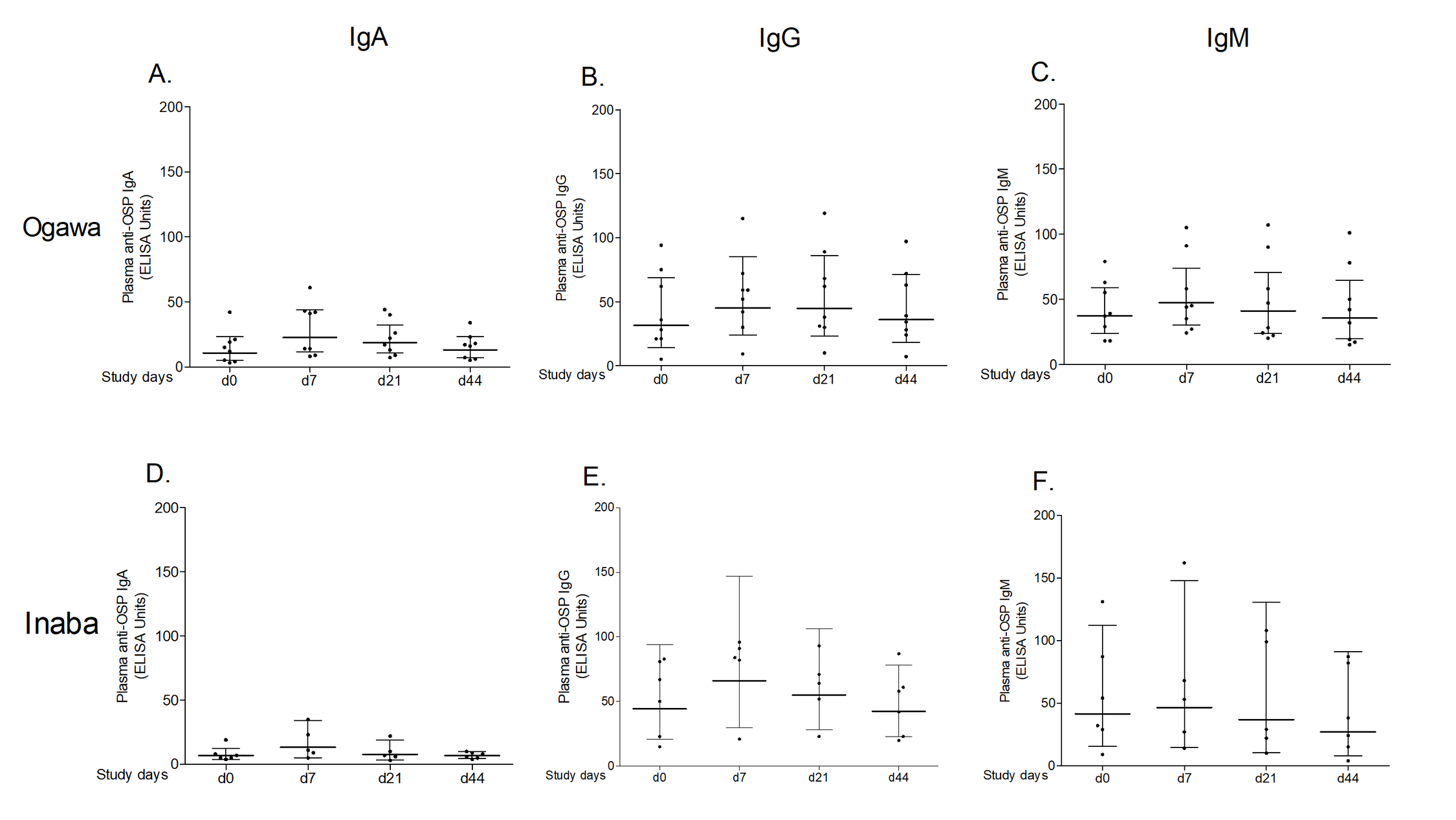

Supplement: S2 Fig — Ogawa OSP responses to (A) IgA, (B) IgG, and (C) IgM and Inaba OSP responses to (D) IgA, (E) IgG, and (F) IgM. Each single dot indicates an individual OSP ELISA unit; horizontal bars represent geometric mean (GM) and error bars indicate 95% confidence intervals. The Wilcoxon signed-rank test was used for analyses of the data. (TIF) [file pntd.0007634.s002.tif]
